# Identification of Myoferlin, a Potential Serodiagnostic Antigen of Clonorchiasis, *via* Immunoproteomic Analysis of Sera From Different Infection Periods and Excretory-Secretory Products of *Clonorchis sinensis*

**DOI:** 10.3389/fcimb.2021.779259

**Published:** 2021-10-18

**Authors:** Xiao-Xiao Ma, Yang-Yuan Qiu, Zhi-Guang Chang, Jun-Feng Gao, Rui-Ruo Jiang, Chun-Lin Li, Chun-Ren Wang, Qiao-Cheng Chang

**Affiliations:** ^1^ School of Public Health, Shantou University, Shantou, China; ^2^ College of Animal Science and Veterinary Medicine, Heilongjiang Bayi Agricultural University, Daqing, China; ^3^ Key Laboratory of Zoonosis Research, Ministry of Education, Institute of Zoonosis, College of Veterinary Medicine, Jilin University, Changchun, China; ^4^ The Seventh Affiliated Hospital, Sun Yat-sen University, Shenzhen, China; ^5^ Institute of NBC Defence, PLA Army, Beijing, China

**Keywords:** *Clonorchis sinensis*, ESPs, Co-IP, myoferlin, diagnosis

## Abstract

Clonorchiasis, which is caused by *Clonorchis sinensis*, is an important foodborne disease worldwide. The excretory-secretory products (ESPs) of *C. sinensis* play important roles in host-parasite interactions by acting as causative agents. In the present study, the ESPs and sera positive for *C. sinensis* were collected to identify proteins specific to the sera of *C. sinensis* (i.e., proteins that do not cross-react with *Fasciola hepatica* and *Schistosoma japonicum*) at different infection periods. Briefly, white Japanese rabbits were artificially infected with *C. sinensis*, and their sera were collected at 7 days post-infection (dpi), 14 dpi, 35 dpi, and 77 dpi. To identify the specific proteins in *C. sinensis*, a co-immunoprecipitation (Co-IP) assay was conducted using shotgun liquid chromatography tandem-mass spectrometry (LC-MS/MS) to pull down the sera roots of *C. sinensis*, *F. hepatica*, and *S. japonicum*. For the annotated proteins, 32, 18, 39, and 35 proteins specific to *C. sinensis* were pulled down by the infected sera at 7, 14, 35, and 77 dpi, respectively. Three proteins, Dynein light chain-1, Dynein light chain-2 and Myoferlin were detected in all infection periods. Of these proteins, myoferlin is known to be overexpressed in several human cancers and could be a promising biomarker and therapeutic target for cancer cases. Accordingly, this protein was selected for further studies. To achieve a better expression, myoferlin was truncated into two parts, Myof1 and Myof2 (1,500 bp and 810 bp), based on the antigenic epitopes provided by bioinformatics. The estimated molecular weight of the recombinant proteins was 57.3 ku (Myof1) and 31.3 ku (Myof2). Further, both Myof1 and Myof2 could be probed by the sera from rabbits infected with *C. sinensis*. No cross-reaction occurred with the positive sera of *S. japonica*, *F. hepatica*, and negative controls. Such findings indicate that myoferlin may be an important diagnostic antigen present in the ESPs. Overall, the present study provides new insights into proteomic changes between ESPs and hosts in different infection periods by LC-MS/MS. Moreover, myoferlin, as a biomarker, may be used to develop an objective method for future diagnosis of clonorchiasis.

## Introduction

*Clonorchis sinensis* is an important foodborne pathogen that causes clonorchiasis as well as liver and biliary diseases when raw fish with *C. sinensis* metacercariae is consumed (Ju et al., 2009). Juvenile fluke, excysting in the duodenum of the host, migrate to the intrahepatic bile ducts, where they develop into adults and survive for more than one decade. It primarily affects mammals, such as dogs, cats, and humans, and its typical clinical symptoms include jaundice, cholangitis, and biliary obstruction (Qian et al., 2016). Clonorchiasis is also closely related to liver fibrosis, other human hepatobiliary diseases, and cholangiocarcinoma (CCA) (Pak et al., 2017). In 2009, *C. sinensis* was classified as a class I biological carcinogen (Véronique et al., 2009). It is estimated that 15 million people suffer from clonorchiasis, and approximately 200 million people, primarily in East and Southeast Asia, such as China, South Korea, and Vietnam, are at risk of infection (Qian et al., 2016; Tang et al., 2016).

Excretory-secretory products (ESPs), which are released by excretory organs during parasitism, can stimulate the host immune response, play important roles in host-parasite interactions, and provide attractive materials for identifying antigenic candidates and new drug targets (Li et al., 2020). Different sources of worms produce various antigen substances and distinct immune response procedures (Pino et al., 1986). Thus, identifying the proteins in ESPs is crucial for understanding the mechanisms inherent to parasite-induced pathogenesis. The ESPs of *C. sinensis* are highly sensitive and specific antigens for the diagnosis of clonorchiasis (Cho et al., 2020). During the development of *C. sinensis*, complex antigens that can affect the host immune system are secreted. Although the antigenic and pathogenic functions of *C. sinensis* have been investigated for several decades, the components and roles of *C. sinensis* ESPs remain limited (Li et al., 2004). The components of *C. sinensis* ESPs are complex; however, they mainly include 7-8, 26-28, and 34-37 ku proteins, with the range of 26-45 ku playing major roles in the production of antibodies in infected rabbits (Hong et al., 2001; Hong et al., 2002). These antigenic candidates, to a great extent, are yet to be characterized. Therefore, discovering reliable and prognostic markers for clonorchiasis diagnosis is of great importance. The protein components of ESPs in several species, such as *F. gigantica*, *S. japonicum*, *S. mansoni*, and *Paragonimus westermani*, have already been characterized using proteomics approaches based on mass spectrometry (Lee et al., 2006; Guillou et al., 2007; Liu et al., 2009; Huang et al., 2019).

Exploiting proteomic tools can enable the identification of more sensitive and specific serodiagnostic antigens that do not cross-react with other parasites. Hence, in this study, the co-immunoprecipitation (Co-IP) assay was used to pull down three types of serum. The sera of rabbits infected with *C. sinensis* were collected at 7 days post infection (dpi), 14 dpi, 35 dpi, and 77 dpi, and the sera positive for *F. hepatica* and *S. japonicum* were also collected. Immunoprecipitation was assessed and characterized using liquid chromatography-tandem mass spectrometry (LC-MS/MS). The objective of in this study was to identify more sensitive and specific antigenic targets in ESPs, and provide more potential diagnostic antigens for clonorchiasis.

## Materials and Methods

### Parasites and Sera

*C. sinensis* metacercariae were collected from naturally infected *Pseudorasbora parva* in the endemic area of Qiqihar, Heilongjiang province, China. Muscular tissue was digested with artificial digestive juice (1% pepsin-hydrochloric acid, Aladdin, China). White Japanese rabbits were purchased from Yisi Experimental Animal Technology Corporation (Changchun City, Jilin Province, China). Fecal examination was conducted before selection to exclude any prior infection with *C. sinensis*. This study was approved by the Animal Health, Animal Care, and Use Committee of the Heilongjiang Bayi Agricultural University. Twenty rabbits (8-9-month-old) negative for *C. sinensis* were selected and randomly divided into two groups: control group and *C. sinensis*-infected group (n=10 each). Rabbits in the experimental group were infected orally with 500 viable metacercariae, while rabbits in the control group were mock-inoculated with 0.85% w/v NaCl solution without metacercariae. After 18 days of infection, the feces of rabbits were collected for fecal examination. Blood samples from each animal were collected aseptically into tubes without anticoagulant, and at 7, 14, 35, and 77 dpi, sera were separated by centrifugation and preserved at -80°C for further use. Positive sera for *F. hepatica* and *S. japonicum* were both obtained from artificially infected rabbits. The rabbits were orally infected with 40 viable *F. hepatica* metacercariae, and the sera were obtained at 90 dpi, stored at the College of Animal Science and Veterinary Medicine, Heilongjiang Bayi Agricultural University. After 42 dpi, positive sera of *S. japonicum* were acquired from rabbits, which infected with 1000 ± 10 *S. japonicum* cercariae, the sera were provided by the Laboratory Animal Center of Shanghai Veterinary Research Institute, Chinese Academy of Agriculture Sciences.

### Collection and Preparation of *C. sinensis* ESPs

CSESPs were prepared according to standard procedures (Pak et al., 2017). Briefly, *C. sinensis* adults were separated from the bile ducts of infected rabbits, washed three times with PBS (stored at 37°C), and preincubated in Locke’s medium (NaCl 8.9 g, KCl 0.42 g, NaHCO_3_ 0.2 g, and CaCl_2_ 0.24 g (wt/vol) at 37°C and 5% CO_2_ for 1 h. Thereafter, the parasites were transferred to fresh medium and incubated for 48 h; the medium was changed at 6-h intervals. Finally, the collected cultures were centrifuged at 10 000 g for 30 min at 4°C, to obtain the supernatant, filtered (0.22 μm), and stored at -80°C.

### Co-IP

The protein A/G plus-agarose immunoprecipitation kit (Santa Cruz Biotechnology, USA) was used for Co-IP according to the manufacturer’s instructions. Briefly, 200 μL of serum positive and negative (7, 14, 35, and 77 dpi) for *C. sinensis* was added into tubes; rabbit sera positive for *S. japonicum* and *F. hepatica* were handled in the same manner. Protein A/G plus-agarose beads (30 μL) were added to each tube and incubated overnight at 4°C. The beads were pelleted by centrifugation at 1,000 g for 30 s at 4°C. Five hundred micrograms *C. sinensis* ESPs (500 µg) was precleared by incubation with protein A/G plus-agarose beads, and incubated for 1.5 h at 4°C. The pellet, collected by centrifugation at 1,000 × g for 5 min at 4°C, was washed three times with PBS buffer, and resuspended in 50 μL SDS loading buffer. The samples were then boiled at 100°C for 10 min. Ten microliters of the sample was analyzed by SDS-PAGE, and the remaining 40 μL was stored for mass spectrometry identification.

### Trypsin Digestion

The stained protein bands were cut into 1 mm^3^ pieces and washed twice with 200 µL of mass spectrometry (MS) water for 10 min. The gels were detained with 50% acetonitrile (ACN) in 50 mM ammonium bicarbonate (ABC), dehydrated by washing with 100% ACN until the gel turned white, and then washed twice with 200 µL of MS water for 10 min/wash. ACN was added to induce dehydration, which occurred until the colloidal particles turned white. Thereafter, they were vacuum dried for 10 min. Proteins on the gels were treated with 200 µL of 10 mM DTT for 1 h at 37°C and subsequently alkylated with 200 µL of 55 mM iodoacetamide (IAM) for 30 min in the dark. The gels were then washed with digested buffer and treated with ACN, as described above. The suspension was washed with the following solutions: MS water (once), ACN (once), MS water (once), and ACN (once) for 10 min/wash, each experiment group was performed three times.

### Liquid Chromatography-Tandem Mass Analysis

Peptides were separated by liquid chromatography using an Acclaim Pep Map 100 column and an EASY-Spray column on an EASY-NLC 1,000 system. The flow rate was set to 0.600 μL/min, and gradient elution was performed for 88 min. The gradient was generated using mobile phase A, which comprised of 100% ddH_2_O containing 0.1% formic acid, and mobile phase B, which consisted of 100% ACN containing 0.1% formic acid. Label-free mass spectrometry was performed using a QE HF-X mass spectrometer. The scan events were composed of one single full MS scan and a 3 s MS/MS scan dependent on the previous scan data. The spray voltages were set at 3.8 kV, and the heated capillary temperature was 320°C. The parameters of the MS/MS scan were as follows: resolution, 15,000; auto gain control target, under 2 × 10^4^; maximum isolation time, 30 ms; and normalized collision energy, 27%, each experiment group was performed three times.

### Data Analysis

Specific proteins were analyzed using Proteome Discoverer 2.4.1.15. Trypsin was employed as the enzyme, which cleaved after all lysine and arginine residues, with up to two missed cleavages allowed. Carbamidomethylating of cysteine was specified as fixed modification, and protein N-terminal acetylation, oxidation of methionine, and pyro-glutamate formation from glutamine were considered as variable modifications for all groups. The raw file of the mass spectrum was identified and analyzed using the commercial software, Max Quant (Thermo Fisher Scientific, Waltham, MA, USA). The precursor ion mass tolerance was set to 15 ppm and the fragment ion mass tolerance was set to 0.02 Da. All data were searched as a single batch with PSM and protein FDR set to 1% using a target decoy approach. The search parameters were as follows: species, *C. sinensis*; dynamic modification, oxidation; mass tolerance of the precursor ion, ± 15 ppm; fragment ion mass tolerance, ± 0.5 Da; and protein false discovery rate (FDR), 0.01. The maximum number of missed cleavages was two. Kyoto Encyclopedia of Genes and Genomes (KEGG) analysis of the proteins of *C. sinensis* was performed to select the most significant pathway and to analyze the relationship between the abundance of specific proteins of different pathways and the different periods of infection.

### *C. sinensis* Myoferlin cDNA Protein Expression

Cloning of cDNAs encoding myoferlin from *C. sinensis* was achieved using the Uniprot database (protein ID H2KUF2). Further, the bioinformatics, including domains, antigen epitopes, and associated interacting proteins, was analyzed. Ultimately, to achieve a better expression of the protein, it was truncated into two parts: Myof1 (aa 160-aa 660) and Myof2 (aa 600-aa 870). The two coding regions of myoferlin were amplified by polymerase chain reaction (PCR) using the following primers: 5’-GGA TCC ACC ATA AAG GAT GTC CGT CA-3’ and 5’-CTC GAG CAG ACA ATG ACT CGT AGC TCA T-3’ (Cs-Myof1), or 5’-GGA TCC CTA CCA CTA GTA AAA GAG CAC G-3’ and 5’-CTC GAG CAC AAC CAA AAG AAC GAT GTC TC-3’ (Cs-Myof2), the underline represents restriction enzyme cutting sites (*Bam*HI, GGA TCC and *Xho*I, CTC GAG). The resulting DNA was digested, purified, and ligated into the *Bam*HI and *Xho*I cloning sites of the pET32a plasmid designed for expression. Recombinant plasmids (pET32a-CsMyof1 and pET32a-CsMyof2) were transformed into *E. coli* BL21 (DE3) cells, and positive clones were selected. Following induction with 1 mM IPTG, bacterial cells were harvested and lysed by sonication in PBS buffer. The lysate was centrifuged at 12,000 g for 20 min at 4°C, and the supernatant was collected. The fusion protein was purified by gel cutting, frozen, and melted five times using liquid nitrogen repeatedly. After centrifugation at 12,000 g for 20 min at 4°C, the supernatant was collected for further use.

### Western Blot

The purified recombinant CsMyof1 and CsMyof2 were subjected to SDS-PAGE (12% gel) and transferred to a Hybond-C pure NC membranes (Immobilon-P, 0.45 μm; Millipore). The membranes were subsequently blocked with blocking buffer (5% w/v skim milk in PBS-T buffer) overnight at 4°C. The sera of rabbits infected with *C. sinensis*, *S. japonicum*, *F. hepatica*, and the negative sera were used as primary antibodies at a dilution of 1:200; the sera were incubated with membranes for 2 h at 37°C. Thereafter, the membranes were washed three times with PBS-T buffer for 30 min. Further incubation was performed using goat anti-rabbit IgG antibody conjugated with HRP at a dilution of 1:5,000 in blocking buffer for 1 h. After washing five times with PBS-T buffer, the membranes were treated with a diaminobenzidine substrate solution for 10 min.

## Results

### Collection of Sera and ESPs

**Figure 1:**
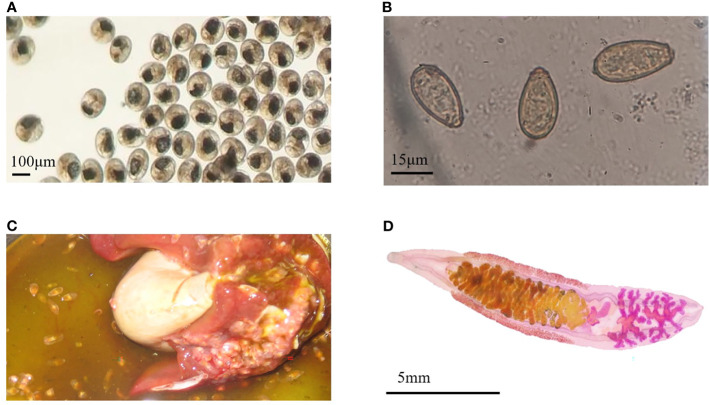
Infection characteristics. **(A)** The Metacercariae of *Clonorchis sinensis* used to infect rabbits. **(B)** *Clonorchis sinensis* eggs observed under an optical microscope. **(C)** Adult worms in the hepatobiliary tract. **(D)** The adult worm collected from the liver of infected rabbit.

The metacercariae of *C. sinensis* were collected from *Pseudorasbora parva* (**Figure 1A**). Fecal examination and morphological examination of eggs were carried out (**Figure 1B**). The first identification was performed at 19 dpi, and all infections were confirmed at 25 dpi. After the infected rabbits were sacrificed, the worms were detected in their hepatobiliary tract (**Figure 1C**); however, no worms were observed in control rabbits. *C. sinensis* was collected and made into films (**Figure 1D**), which complies with its typical characteristics, such as the branched testicles and S-shaped excretory sac. The model of rabbits infected with *C. sinensis* was successfully established, and sera at different periods of infection (7, 14, 35, and 77 dpi) were collected. Adult C. *sinensis* worms were collected and cultured *in vitro* for 48 h to prepare the ESPs. The majority of proteins among the ESPs ranged in molecular weight from 10 to 170 ku (**Figure S1A**). A total of 334 proteins were obtained, among which 254 annotated proteins were obtained from the *C. sinensis* protein library by BLAST homology comparison and Uniport identification (**Figure S1B**).

### LC-MS/MS Analysis and the Identification of Sera Proteins at Different Periods

**Figure 2:**
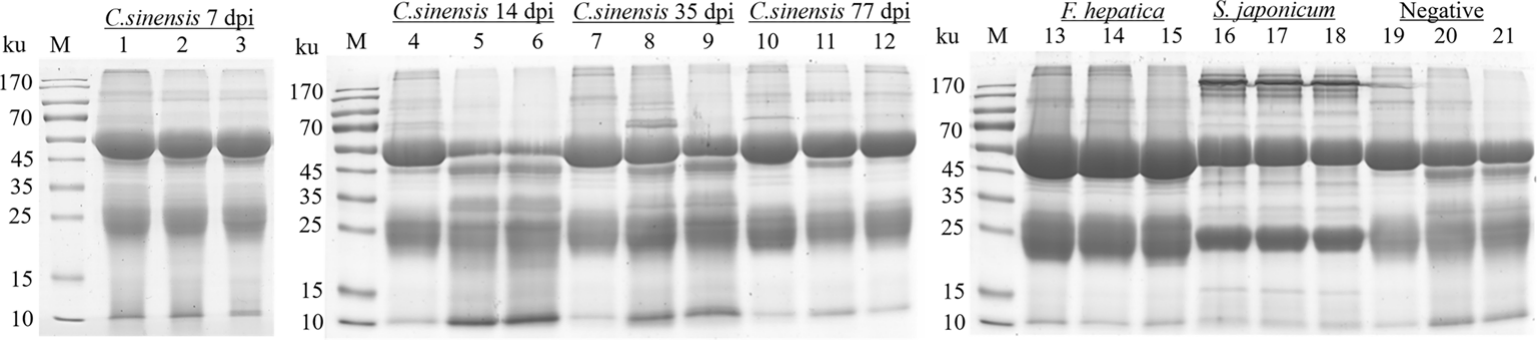
SDS-PAGE analysis of rabbit sera samples of *C. sinensis* at different infection period; *F. hepatica* and *S. japonicum* were co-cultured with the *C. sinensis* ESPs. Lines 1-3: proteins pulled down by rabbit serum at 7 dpi with *C. sinensis*. Lines 4-6: proteins pulled down by rabbit serum at 14 dpi with *C. sinensis*. Lines 7-9: proteins pulled down by rabbit serum at 35 dpi with *C. sinensis*. Lines 10-12: proteins pulled down by rabbit serum at 77 dpi with *C. sinensis*. Lines 13-15: proteins pulled down by *F. hepatica* serum. Lines 16-18: proteins pulled down by *S. japonicum* serum. Lines 19-21: proteins pulled down by rabbit negative serum.

**Figure 3:**
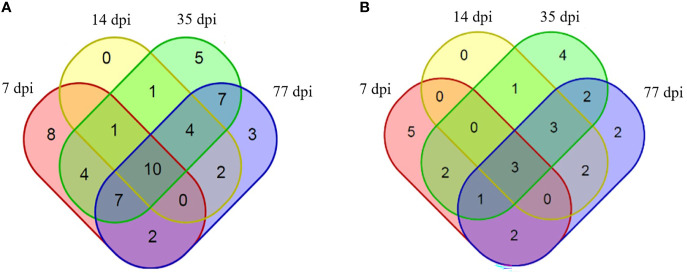
Proteins identified to be binding to rabbit sera at different infection time points. **(A)** Proteins specifically identified to bind to rabbit serum at 7 dpi with *C. sinensis* (pink), 14 dpi (yellow), 35 dpi (green) and 77 dpi (blue), compared with negative serum. **(B)** Proteins specifically identified to bind to rabbit serum at 7 dpi with *C. sinensis* (pink), 14 dpi (yellow), 35 dpi (green), and 77 dpi (blue), compared with *S. japonicum* serum, *F. hepatica* serum, and negative serum.

An immuno-proteomic approach was used to identify the proteins secreted by *C. sinensis*, specifically using infection sera to pull down the *C. sinensis* ESPs that might be involved in host-parasite interactions. The results of the Co-IP assay revealed that the antibodies from serum at the periods of 7, 14, 35, and 77 dpi, could recognize and pull down the specific proteins from *C. sinensis* ESPs, with most proteins ranging in molecular weight from 15 to 170 ku (**Figure 2**). There were 32, 18, 39, and 35 proteins in the first round of differential screening between positive and negative serum samples (**Figure 3A**). The second round of screening was carried out by comparing and analyzing different types of positive sera, including *F. hepatica*, *S. japonicum*, and *C. sinensis*, based on the first screening (**Figure 3B**). According to the LC-MS/MS analysis, the obtained data was compared with the *C. sinensis* protein library to screen the differential proteins in each period. There were 13, 9, 16, and 15 types of proteins specific to the periods of 7, 14, 35, and 77 dpi with *C. sinensis*, respectively (**Tables 1**–**4**). Five proteins could only be detected in the early stage (7 dpi), which may explain their induction of an early response in the host, and three proteins were found to be specifically co-purified in all four periods of *C. sinensis* (**Table 5**), including dynein light chain-1, dynein light chain-2, and myoferlin, which were characterized by label-free quantification and functional analysis. These three proteins are involved in body movement and energy metabolism, and were found to display high expression at different stages, thereby providing energy for *C. sinensis*. Myoferlin, an important protein related to tumors, is a member of the ferlin family and is a type II transmembrane protein with a single transmembrane domain at the C terminus.

**Table 1:** *Clonorchis sinensis* excretory and secretory products which were detected by sera of rabbits in 7 dpi.

Accession	Species	Description	Peptides	Unique Peptides	Coverage	MW [ku]	pI
G7YYI1	*C.sinensis*	NADH pyrophosphatase	1	1	5	21.7	5.8
A0A3R7D3C8	*C.sinensis*	Beta-galactosidase	1	1	1	100.9	6.32
G7YV19	*C.sinensis*	Uncharacterized protein	1	1	2	40.8	9.44
G7YFQ8	*C.sinensis*	Cleavage and polyadenylation specificity factor subunit 3	1	1	1	77.9	6.81
A0A3R7FK18	*C.sinensis*	Sorcin	1	1	3	46.7	8.85
A0A3R7CX34	*C.sinensis*	Dynein light chain-1	1	1	8	19.3	5.26
H2KUF2	*C.sinensis*	Myoferlin	1	1	1	105.3	6.02
G7YYI0	*C.sinensis*	NAD (+) kinase	2	2	9	32.5	6.79
A0A3R7GD73	*C.sinensis*	Dynein light chain-2	3	3	38	10.5	8.1
A0A3R7C7M8	*C.sinensis*	ATP synthase subunit alpha	1	1	1	102.9	9.54
G7YYJ7	*C.sinensis*	Acetylornithine deacetylase	2	2	14	29.1	6.13
O96912	*C.sinensis*	Cysteine proteinase	2	2	18	19.8	5.95
G7YFI6	*C.sinensis*	Putative cys1 protein	10	2	6	185.3	7.25

**Table 2:** *Clonorchis sinensis* excretory and secretory products which were detected by sera of rabbits in 14 dpi.

Accession	Species	Description	Peptides	Unique Peptides	Coverage (%)	MW [ku]	pI
G7YBN0	*C. sinensis*	Charged multivesicular body protein 2A	1	1	2	58.3	9.54
H2KUQ9	*C. sinensis*	Serpin B	1	1	3	37.9	5.2
H2KUG0	*C. sinensis*	Universal stress protein	1	1	8	18.7	6.86
H2KPA8	*C. sinensis*	DNA damage	1	1	5	28	6.14
A0A3R7CX34	*C. sinensis*	Dynein light chain-1	1	1	8	19.3	5.26
H2KUF2	*C. sinensis*	Myoferlin	1	1	1	105.3	6.02
G7YL60	*C. sinensis*	Phospholipid scramblase	1	1	9	16	6.87
A0A3R7GD73	*C. sinensis*	Dynein light chain-2	3	3	38	10.5	8.1
A0A419PK16	*C. sinensis*	Adenosylhomocysteinase	2	2	6	47.7	6.14

**Table 3:** *Clonorchis sinensis* excretory and secretory products which were detected by sera of rabbits in 35 dpi.

Accession	Species	Description	Peptides	Unique Peptides	Coverage (%)	MW [ku]	pI
G7YJJ5	*C. sinensis*	Transient receptor potential cation channel subfamily M member 2	1	1	1	226.5	6.58
G7YYH0	*C. sinensis*	Phosphomethylpyrimidine synthase	1	1	2	48.9	5.29
G7YG42	*C. sinensis*	16 kDa calcium-binding protein	1	1	7	17.4	4.82
H2KUG0	*C. sinensis*	Universal stress protein	1	1	8	18.7	6.86
A0A3R7FK18	*C. sinensis*	Sorcin	1	1	3	46.7	8.85
H2KPA8	*C. sinensis*	DNA damage-regulated autophagy modulator protein 2	1	1	5	28	6.14
G7YCE6	*C. sinensis*	Ras-related protein Ral-A	1	1	2	66.9	8.72
A0A3R7CX34	*C. sinensis*	Dynein light chain-1	1	1	8	19.3	5.26
H2KUF2	*C. sinensis*	Myoferlin	1	1	1	105.3	6.02
G7YL60	*C. sinensis*	Phospholipid scramblase	1	1	9	16	6.87
A0SWW1	*C. sinensis*	Glutathione peroxidase	2	2	10	19.5	7.44
A0A3R7GD73	*C. sinensis*	Dynein light chain-2	3	3	38	10.5	8.1
G7YVN8	*C. sinensis*	Proactivator polypeptide	1	1	2	70.9	9.1
A0A419PK16	*C. sinensis*	Adenosylhomocysteinase	2	2	6	47.7	6.14
A0A3R7C7M8	*C. sinensis*	ATP synthase subunit alpha	1	1	1	102.9	9.54
G7YFI6	*C. sinensis*	Putative cys1 protein	10	2	6	185.3	7.25

**Table 4:** *Clonorchis sinensis* excretory and secretory products which were detected by sera of rabbits in 77 dpi.

Accession	Species	Description	Peptides	Unique Peptides	Coverage (%)	MW [ku]	pI
G7YBN0	*C. sinensis*	Charged multivesicular body protein 2A	1	1	2	58.3	9.54
A0A3R7D3C8	*C. sinensis*	Beta-galactosidase	1	1	1	100.9	6.32
G7YYH0	*C. sinensis*	Phosphomethylpyrimidine synthase	1	1	2	48.9	5.29
H2KUQ9	*C. sinensis*	Serpin B	1	1	3	37.9	5.2
G7YG42	*C. sinensis*	16 kDa calcium-binding protein	1	1	7	17.4	4.82
H2KUG0	*C. sinensis*	Universal stress protein	1	1	8	18.7	6.86
A0A3R7FK18	*C. sinensis*	Sorcin	1	1	3	46.7	8.85
H2KPA8	*C. sinensis*	DNA damage-regulated autophagy modulator protein 2	1	1	5	28	6.14
A0A3R7CX34	*C. sinensis*	Dynein light chain-1	1	1	8	19.3	5.26
H2KUF2	*C. sinensis*	Myoferlin	1	1	1	105.3	6.02
B5G4Y2	*C. sinensis*	Aspartic protease	2	2	5	46.6	7.33
G7YL60	*C. sinensis*	Phospholipid scramblase	1	1	9	16	6.87
A0A3R7GD73	*C. sinensis*	Dynein light chain-2	3	3	38	10.5	8.1
H2KTR5	*C. sinensis*	Fibropellin-1	4	1	2	210.4	7.34
O96912	*C. sinensis*	Cysteine proteinase	2	2	18	19.8	5.95

**Table 5:** *Clonorchis sinensis* excretory and secretory products which were detected by rabbit post-infection in all four periods.

Accession	Species	Description	Peptides	Unique Peptides	Coverage (%)	MW [ku]	pI
A0A3R7CX34	*C. sinensis*	Dynein light chain-1	1	1	8	19.3	5.26
H2KUF2	*C. sinensis*	Myoferlin	1	1	1	105.3	6.02
A0A3R7GD73	*C. sinensis*	Dynein light chain-2	3	3	38	10.5	8.1

### The Selected Signal Path Based on KEGG

**Figure 4:**
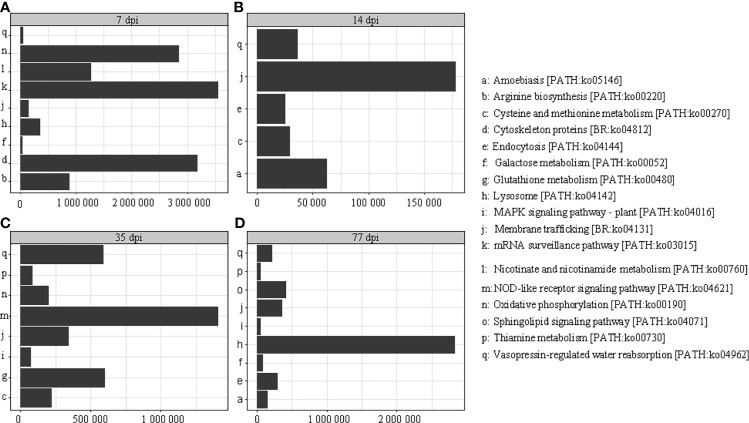
Kyoto Encyclopedia of Genes and Genomes (KEGG) analysis of the different periods after infection; the different periods are displayed on the horizontal axis and the abundance expressed by specific proteins is displayed on the longitudinal axis. **(A)** KEGG analysis of proteins at 7 dpi with *C. sinensis*. **(B)** KEGG analysis of proteins at 14 dpi with *C. sinensis*. **(C)** KEGG analysis of proteins at 35 dpi with *C. sinensis*. **(D)** KEGG analysis of proteins at 77 dpi with *C. sinensis*.

Twenty-seven proteins were detected in *C. sinensis*, but not in *F. hepatica* and *S. japonicum*, where they participate in host-parasite interactions. KEGG analysis revealed that thease proteins were mainly involved in oxidative phosphorylation, membrane transport, signal transduction, and metabolism. The abundance of proteins was different in each period of infection; cystoskeleton, oxidative phosphorylation, and mRNA surveillance pathway-related proteins were highly expressed at the early infection stage (7 dpi), and may be involved in early immune evasion (**Figure 4A**). In the middle stage of parasite infection (14 dpi), membrane trafficking related proteins were evidently increased, and may play important roles in providing energy for parasites (**Figure 4B**). NOD-like receptor signaling pathway, glutathione metabolism, and lysosome-related proteins were highly expressed at the late infection stage (35 dpi and 77 dpi); lysosomes secreted by *C. sinensis* can effectively inactivate the proteins released by hosts to protect themselves (**Figures 4C, D**).

### Analysis of the Amino Acid Sequence of Myoferlin

Myoferlin has two same domains: protein kinase C conserved region 2 (aa415-aa514, aa655-aa783). Regions with significant homology to the C2 domain have been identified in many proteins. The C2 domain is thought to be involved in calcium-dependent phospholipid binding and membrane-targeting processes, such as subcellular localization. Myoferlin also has two low-complexity domains (domain I, aa254-aa265 and II aa388-aa399) and a transmembrane region (aa887-aa909) (**Figure S2A**). The B cell epitopes of Myoferlin were predicted *via* IEDB (http://tools.iedb.org/), which resulted in ten linear epitopes (**Table S1**) and five discontinuous epitopes (**Table S2**). The predicted scores were all greater than 0.5, which indicated that the protein has a potential for binding antigens. The protein network interactions of myoferlin were also predicted (https://string-db.org/), which resulted in a total of 11 associated proteins (**Figure S2B**). Among these relative proteins, ATPase (ASAN1) is required for the post-translational delivery of tail-anchored proteins to the endoplasmic reticulum. ATPase also recognizes and selectively binds to the transmembrane domain of TA proteins in the cytosol. Vesicle-associated membrane protein 2 (VAMP2), a member of the SNARE family, is the first synaptobrevin studied in synaptic vesicles, and is regarded as a molecule that plays a key role in the process of cell growth, neurotransmission, hormone secretion, and insulin-dependent glucose uptake.

### Gene Cloning and Antigenicity Analysis of Recombinant Proteins

**Figure 5:**
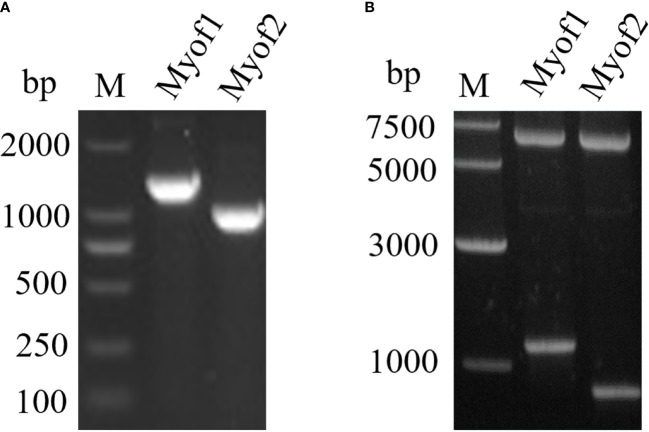
Amplification and cloning of Myof1 and Myof2. **(A)** The amplified genes of Myof1 and Myof2 (1500 bp and 810 bp) using specific oligonucleotide primers. **(B)** Cloning of the coding sequence for Myof1 and Myof2 into the pET32a vector; the inserted DNA was digested with *Bam*HI and *Xho*I.

**Figure 6:**
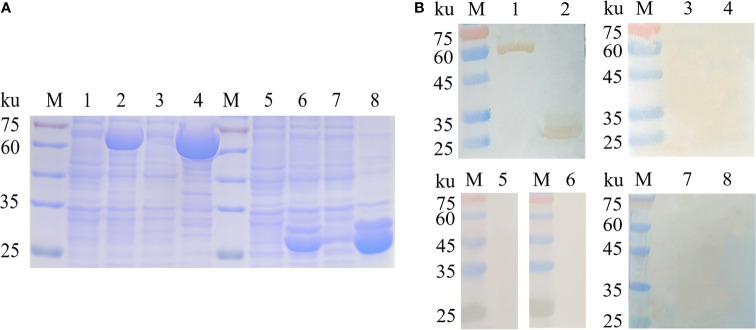
Expression and antigenicity analysis of Myof1 and Myof2. **(A)** The recombinant proteins, Myof1 and Myof2, were subjected to SDS-PAGE (12% gel). M: standard molecular size; Line 1: Un-induced recombinant Myof1-C/BL21; Line 2: Induced recombinant pET-32a-Myof1-C/BL21 with IPTG; Lines 4-5: Supernatant and precipitate of the expression product from the recombinant pET-32a-Myof1 with IPTG induction, respectively. Line 5: Un-induced recombinant Myof2-C/BL21; Line 6: Induced recombinant pET-32a-Myof2-C/BL21 with IPTG; Lines 7-8: Supernatant and precipitate of the expression product from the recombinant pET-32a-Myof2 with IPTG induction, respectively. **(B)** The recombinant proteins reacted with different sera. M: standard molecular size; Lines 1-2: The recombinant proteins, Myof1 and Myof2, incubated with positive sera of the rabbit infected with *C sinensis*; Lines 3-4: Negative serum of rabbit as a control. Lines 5-6: The recombinant proteins, Myof1 and Myof2, incubated with positive sera of the rabbit infected with *S. japonicum*. Lines 7-8: The recombinant proteins, Myof1 and Myof2, incubated with positive sera of the rabbit infected with *F. hepatica*.

To achieve a better expression, myoferlin was truncated into two parts (Myof1 and Myof2) according to the antigenic epitopes provided by bioinformatics. The Myof1 and Myof2 (1,500 bp and 810 bp) genes were amplified using specific oligonucleotide primers (**Figure 5A**) and sub-cloned into the *E. coli* pET32a expression vector. After sub-cloning, the size of the inserted DNA was confirmed by restriction digestion with *Bam*HI and *Xho*I (**Figure 5B**). Subsequently, the cloned Myof1 and Myof2 were successfully expressed in *E. coli* BL21 (DE3) cells. The recombinant proteins were purified by gel cutting and analyzed by SDS-PAGE (**Figure 6A**). The molecular weights of the recombinant proteins were determined to be approximately 60 ku (Myof1) and 31 ku (Myof2). The sera from rabbits infected with *C. sinensis* could be probed at different levels, and the sera positive for *S. japonica*, *F. hepatica*, and from naive rabbit could hardly be probed (**Figure 6B**), which indicates that myoferlin may be a potential antigen for the detection of clonorchiasis.

## Discussion

Clonorchiasis, a foodborne trematodiasis, is an emerging public health problem in China, Korea, and Vietnam (Na et al., 2020). Direct parasite irritation in the bile duct epithelium can cause not only mechanical irritation, but also chemical impairment. Epidemiological and experimental studies have reported that *C. sinensis* infections can induce biliary epithelial hyperplasia, periductal fibrosis, and cystic changes in the ducts, and may also facilitate the development of cholangiocarcinoma (Qian et al., 2016). ESPs, consisting of a complex mixture of proteins, carbohydrates, and lipids, are generally considered to play important roles in host-parasite interactions, including invasion, digestion, detoxification, and immune evasion (Kim et al., 2021). *C. sinensis* continuously releases ESPs (complex mixture of proteins, carbohydrates, lipids, etc.) from the excretory orifice to the outside. Cells exposed to ESPs exert a variety of pathophysiological reactions, including proliferation, apoptosis, destruction of redox homeostasis, and inflammation (Serradell et al., 2007; Kim et al., 2008a). The transcriptomic and proteomic profiles of human cholangiocarcinoma cells treated with ESPs demonstrated that host mRNA was upregulated or downregulated to varying degrees; upregulated genes were related to tumorigenesis, cell proliferation, and differentiation, while downregulated genes were related to apoptosis (Pak et al., 2014). Identification of proteins in ESPs is thus crucial to our understanding of the mechanisms underlying parasite-induced pathogenesis. Proteomic analysis of *C. sinensis* ESPs revealed that the identified proteins have high immunogenicity and certain specificity, such as detoxifying enzymes, myoglobin proteases, and grain-like growth factors (Mulvenna et al., 2010). Cathepsin F is the major protein in ESPs at 0-3 h, and the main protein of enzymatic proteolytic activity (Kang et al., 2010). ESPs at 0-5 h regulate the proliferation and apoptosis of cholangiocarcinoma cells (Kim et al., 2008b). Cysteine, ESPs at 0-12 h, induce cytotoxicity (Pak et al., 2009). ESPs from *C. sinensis* are directly exposed to the host immune system and are widely used as antigens in serological assays. Further, the antigens of ESPs tend to be superior to those of crude extracts in *C. sinensis* for serodiagnosis of clonorchiasis. Methionine aminopeptidase 2 acid phosphatase, and fructose-1,6-bisphosphatase were found to display high specificity and sensitivity, indicating that they are potential diagnostic antigens (Zheng et al., 2011; Zheng et al., 2013).

Although many studies have revealed the components of ESPs at different periods of *C. sinensis*, few studies have examined the relationship between ESPs and sera at different infection periods. Such finding can not only help us identify the key proteins that interact with sera, but also establish an early diagnosis method for clonorchiasis. Five proteins were found in this study, which were only present in the7 dpi interacting with ESPs. Among them, the cleavage and polyadenylation specificity factor proteins exist widely in many organisms (Shi and Manley, 2015). Most mRNA precursors (pre-mRNAs) are cleaved and polyadenylated at the 3’ end prior to their export from the nucleus (Sun et al., 2020). This sequence of events is carefully orchestrated, providing both a tight regulation of cleavage/polyadenylation and the opportunity to select from multiple cleavage sites with various affinities (Gruber and Zavolan, 2019). Switching between these sites can lead to changes in the length and sequence of the 3’ UTR of mRNAs, which has many effects on protein expression, mRNA stability, and localization (El Mouali and Balsalobre, 2019). Acetylornithine deacetylase is mainly involved in the ornithine cycle, and the formation of N-acetyl-glutamate from glutamate and acetyl-CoA in a reaction catalyzed by N-acetyl-glutamate synthase (Javid-Majd and Blanchard, 2000). NOD-like receptors are a large family of 22 intracellular proteins in humans that perform a diverse array of cellular functions and play key roles in the regulation of innate immune responses (Lupfer et al., 2020).

To date, ESPs have been known to contain sensitive antigens for the diagnosis of clonorchiasis, and popular antigenic candidates are yet to be characterized in detail. In this study, proteomic and Co-IP assays were used to pull down three types of serum: *C. sinensis*, *F. hepatica*, and *S. japonicum* interacting with the ESPs of *C. sinensis*. Proteins specific to *C. sinensis* were identified by cross-screening using shotgun LC-MS/MS. Due to the limitations of the existing database, only 254 of the 334 proteins were annotated in the total mass spectrum of ESPs. We identified multiple proteins from positive serum samples of *C. sinensis* at different infection periods, and the functions of the identified proteins were classified based on various biological processes. Protein relative pathway analysis revealed that these proteins were mainly involved in oxidative phosphorylation, signal transduction, and metabolism, thereby playing important roles in the interaction between parasites and hosts. Among these proteins, we focused on Myoferlin, one of the three proteins detected in the serum collected at the four infection periods. Myoferlin, which belongs to the ferlin family, is an evolutionarily conserved family of vesicle fusion proteins that is reported to be involved in myoblast fusion, vesicle trafficking, and plasma membrane integrity (Turtoi et al., 2013). Recent studies have shown that myoferlin is overexpressed in several human cancers and enhances tumor progression by regulating migration, invasion, and tumorigenesis (Zhang et al., 2018). Myoferlin is highly involved in oxaliplatin resistance and tumor progression in gastric cancer. The protein could be a promising biomarker and a therapeutic target for cases of oxaliplatin-resistant gastric cancer (Gupta et al., 2021). Studies have shown that myoferlin is also involved in pivotal physiological functions related to numerous cell membranes, such as extracellular secretion, endocytosis, vesicle trafficking, membrane repair, membrane receptor recycling, and secreted protein efflux (Gu et al., 2020).

However, the function of myoferlin in clonorchiasis, especially its effects on detection sensitivity, has received little attention. Thus, we cloned and expressed both Myof1 and Myof2 of the protein to determine their reactogenicity. According to the cross-reactivity analysis results, both Myof1 and Myof2 did not show any cross-reactivity with the sera from *F. hepatica*, *S. japonicum*, and uninfected rabbits. Further, both parts of myoferlin demonstrated a higher degree of specificity to the sera of infected rabbits. Our findings indicate that the immuno-proteomic approaches used in this study could have a significant effect on the identification of serodiagnostic antigens against clonorchiasis. Additionally, myoferlin may be a potential protein for the immunological diagnosis of clonorchiasis.

## Data Availability Statement

The datasets presented in this study can be found in online repositories. The names of the repository/repositories and accession number(s) can be found below: http://www.proteomexchange.org/, PXD028236.

## Ethics Statement 

The animal study was reviewed and approved by Animal Health, Animal Care and Use Committee of Heilongjiang Bayi Agricultural University.

## Author Contributions

C-RW and Q-CC designed the project and experiments. X-XM and Y-YQ conducted the experiments. Z-GC and R-RJ analyzed the data. C-LL and J-FG made the images. X-XM, Y-YQ and Q-CC prepared the manuscript. All authors contributed to the article and approved the submitted version.

## Funding

This work was supported by the National Natural Science Foundation of China (Grant Nos. 31672399, 81802869 and 32172886), Heilongjiang Postdoctoral Science Foundation (Grant No. LBH-Z19191), and the Heilongjiang Provincial Postdoctoral Natural Science Foundation of China (Grant No. LH2021C071).

## Conflict of Interest

The authors declare that the research was conducted in the absence of any commercial or financial relationships that could be construed as a potential conflict of interest.

## Publisher’s Note

All claims expressed in this article are solely those of the authors and do not necessarily represent those of their affiliated organizations, or those of the publisher, the editors and the reviewers. Any product that may be evaluated in this article, or claim that may be made by its manufacturer, is not guaranteed or endorsed by the publisher.

## Supplementary Material

Supplementary Figure 1 Mass spectrometry analysis of the ESP components. **(A)** SDS-PAGE analysis of the total ESPs of *C. sinensis*. **(B)** Comparative analysis between the identified proteins and *C. sinensis* protein library.

Supplementary Figure 2 Domains and interacting proteins prediction of myoferlin. **(A)** The domains predetermination of myoferlin. **(B)** The protein network interaction predetermination of myoferlin.
