# Supplementary figures and images for: Identification of Myoferlin, a Potential Serodiagnostic Antigen of Clonorchiasis, via Immunoproteomic Analysis of Sera From Different Infection Periods and Excretory-Secretory Products of Clonorchis sinensis
Source: Front Cell Infect Microbiol. 2021 Oct 18;11:779259. doi: 10.3389/fcimb.2021.779259 (PMC8558468; doi:10.3389/fcimb.2021.779259)

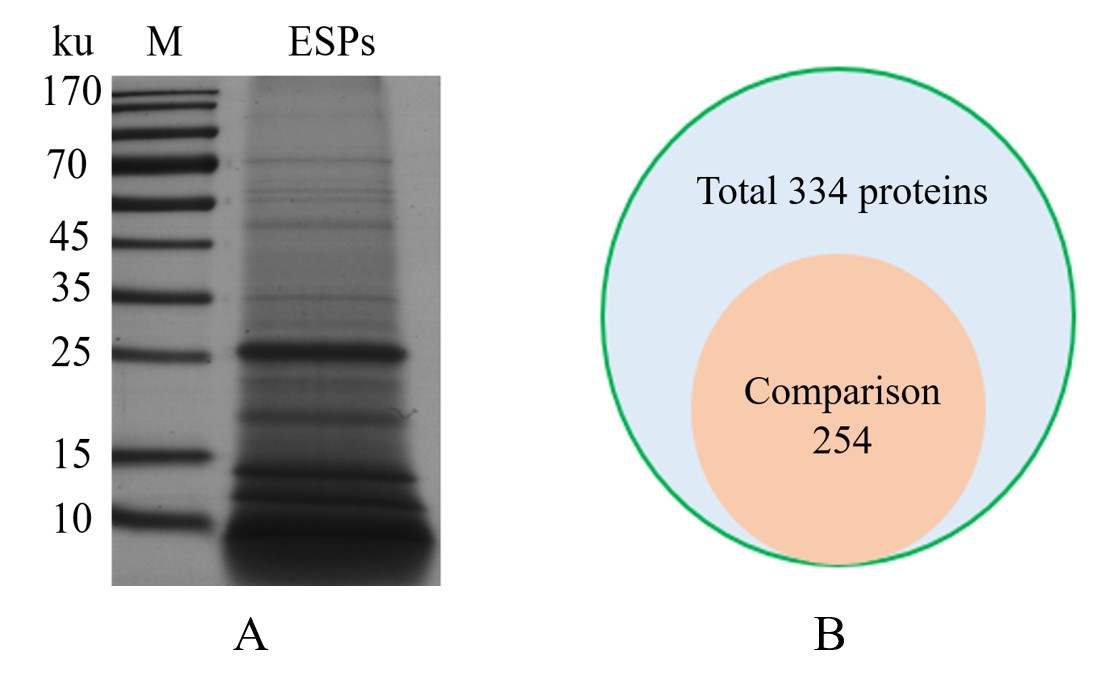

Supplement: Supplementary Figure 1 — Mass spectrometry analysis of the ESP components. (A) SDS-PAGE analysis of the total ESPs of C. sinensis. (B) Comparative analysis between the identified proteins and C. sinensis protein library. [file Image_1.jpeg]

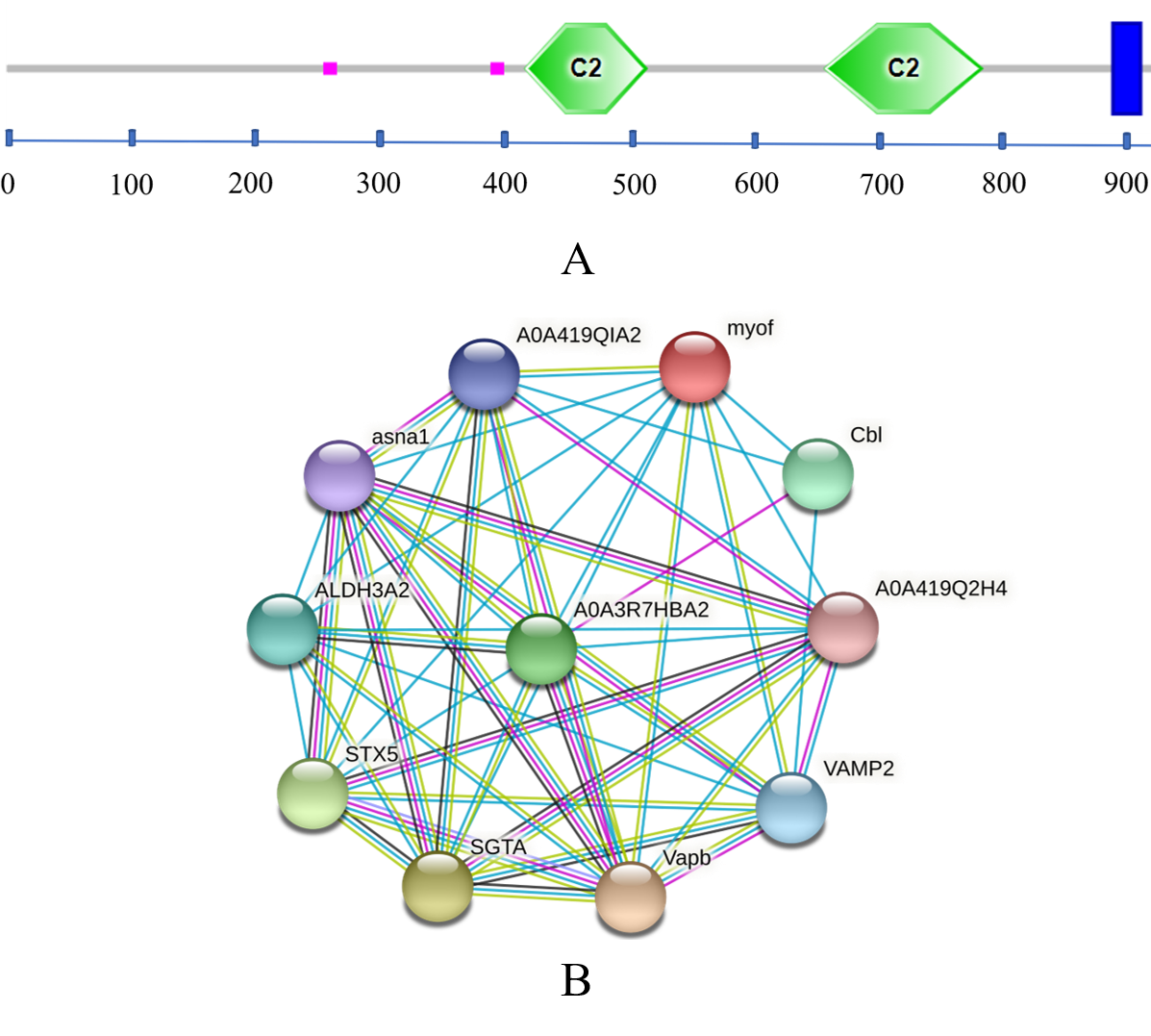

Supplement: Supplementary Figure 2 — Domains and interacting proteins prediction of myoferlin. (A) The domains predetermination of myoferlin. (B) The protein network interaction predetermination of myoferlin. [file Image_2.jpeg]
