# Supplementary material for: Identification of Myoferlin, a Potential Serodiagnostic Antigen of Clonorchiasis, via Immunoproteomic Analysis of Sera From Different Infection Periods and Excretory-Secretory Products of Clonorchis sinensis
Source: Front Cell Infect Microbiol. 2021 Oct 18;11:779259. doi: 10.3389/fcimb.2021.779259 (PMC8558468; doi:10.3389/fcimb.2021.779259)
Supplement: Supplementary file 3 [file Table_1.docx]

Table S1 The predicted linear epitopes of myoferlin

| No. | Start | End | Peptide | Number of residues | Score |
| --- | --- | --- | --- | --- | --- |
| 1 | 631 | 657 | DLFPVNLGEPGPAVDVSPRRPNEYELR | 27 | 0.834 |
| 2 | 501 | 529 | DLENRRLSKYRATCGLPQTYCTSGPTQWR | 29 | 0.785 |
| 3 | 568 | 613 | QKNFMLEHFERGTQSNPHLGGPKERLALHILNHLPLVKEHVETRLL | 46 | 0.739 |
| 4 | 412 | 423 | EECVVRIYVIRA | 12 | 0.703 |
| 5 | 547 | 560 | LPPPQYEEPSEATP | 14 | 0.687 |
| 6 | 452 | 478 | DKYVPNTLNPEFGCLFQMKCLLPVEKD | 27 | 0.634 |
| 7 | 711 | 745 | WRFVFPFFYLPAENMMVIKRKEHFWSLDTTERRVR | 35 | 0.605 |
| 8 | 689 | 694 | GVDERQ | 6 | 0.599 |
| 9 | 756 | 760 | DLFSA | 5 | 0.554 |
| 10 | 430 | 435 | DASGLA | 6 | 0.524 |
