# Supplementary material for: Identification of Myoferlin, a Potential Serodiagnostic Antigen of Clonorchiasis, via Immunoproteomic Analysis of Sera From Different Infection Periods and Excretory-Secretory Products of Clonorchis sinensis
Source: Front Cell Infect Microbiol. 2021 Oct 18;11:779259. doi: 10.3389/fcimb.2021.779259 (PMC8558468; doi:10.3389/fcimb.2021.779259)
Supplement: Supplementary file 4 [file Table_2.docx]

Table S2 The predicted discontinuous epitopes of myoferlin

| No. | Peptide | Number of residues | Score |
| --- | --- | --- | --- |
| 1 | A:D631, A:L632, A:F633, A:P634, A:V635, A:N636, A:L637, A:G638, A:E639, A:P640, A:G641, A:P642, A:A643, A:V644, A:D645, A:V646, A:S647, A:P648, A:R649, A:R650, A:P651, A:N652, A:E653, A:Y654, A:E655, A:R657, A:V714, A:F715, A:P716, A:F717, A:F718, A:Y719, A:L720, A:P721, A:A722, A:E723, A:N724, A:M725, A:M726, A:V727, A:I728, A:R730 | 42 | 0.778 |
| 2 | A:E553, A:E554, A:P555, A:S556, A:E557, A:A558, A:T559, A:P560, A:S561, A:R563, A:C564, A:R565, A:K569, A:M572, A:L573, A:E574, A:H575, A:F576, A:E577, A:R578, A:G579, A:T580, A:Q581, A:S582, A:N583, A:P584, A:H585, A:L586, A:G587, A:G588, A:P589, A:K590, A:E591, A:L593, A:A594, A:L595, A:H596, A:I597, A:L598, A:N599, A:H600, A:L601, A:P602, A:L603, A:V604, A:K605, A:E606, A:H607, A:V608, A:E609, A:T610, A:R611, A:L612, A:L613 | 54 | 0.726 |
| 3 | A:L547, A:P548, A:P549, A:P550, A:Q551, A:Y552 | 6 | 0.712 |
| 4 | A:E412, A:E413, A:C414, A:V415, A:V416, A:R417, A:I418, A:Y419, A:I421, A:R422, A:A423, A:I424, A:P428, A:D430, A:A431, A:S432, A:G433, A:L434, A:A435, A:D436, A:L443, A:G444, A:S450, A:D452, A:V455, A:P456, A:N457, A:T458, A:L459, A:N460, A:P461, A:E462, A:F463, A:G464, A:C465, A:L466, A:F467, A:Q468, A:M469, A:K470, A:C471, A:L472, A:L473, A:P474, A:V475, A:E476, A:K477, A:D478, A:D501, A:L502, A:E503, A:N504, A:R505, A:L507, A:S508, A:K509, A:Y510, A:R511, A:A512, A:T513, A:C514, A:G515, A:L516, A:P517, A:Q518, A:T519, A:Y520, A:C521, A:T522, A:S523, A:G524, A:P525, A:T526, A:Q527, A:R529 | 75 | 0.677 |
| 5 | A:G689, A:V690, A:D691, A:E692, A:R693, A:K729, A:K731, A:E732, A:F734, A:W735, A:S736, A:L737, A:D738, A:T739, A:T740, A:E741, A:R743, A:V744, A:R745 | 19 | 0.599 |
